# Clinical Efficacy of Serum Antiglycopeptidolipid Core IgA Antibody Test for Screening Nontuberculous Mycobacterial Pulmonary Disease in Bronchiectasis: A European Multicenter Cohort Study

**DOI:** 10.1016/j.chest.2024.10.029

**Published:** 2024-10-28

**Authors:** Hayoung Choi, Chloe Hughes, Zsofia Eke, Morven Shuttleworth, Michal Shteinberg, Eva Polverino, Pieter C. Goeminne, Tobias Welte, Francesco Blasi, Amelia Shoemark, Merete B. Long, Stefano Aliberti, Charles S. Haworth, Felix C. Ringshausen, Michael R. Loebinger, Natalie Lorent, James D. Chalmers

**Affiliations:** a Division of Pulmonary, Allergy, and Critical Care Medicine, Department of Internal Medicine, Hallym University Kangnam Sacred Heart Hospital, Seoul, Korea; b Division of Molecular and Clinical Medicine, University of Dundee, Ninewells Hospital and Medical School, Dundee, Scotland; c Cambridge Centre for Lung Infection, Royal Papworth Hospital and University of Cambridge, Cambridge; d Host Defence Unit, Department of Respiratory Medicine, Royal Brompton Hospital and Harefield NHS Foundation Trust, Imperial College London, London, England; e National Heart and Lung Institute, Imperial College London, London, England; f Pulmonology Institute and CF Center, Carmel Medical Center and the Technion - Israel Institute of Technology, B. Rappaport Faculty of Medicine, Haifa, Israel; g Hospital Clinic of Barcelona, University of Barcelona, CIBERES, IDIBAPS, Barcelona, Spain; h Department of Respiratory Disease, AZ Nikolaas, Sint-Niklaas, Belgium; i Department of Respiratory Diseases, University Hospitals Leuven, Belgium; j Department of Chronic Diseases, Metabolism and Aging, BREATHE Laboratory, KU Leuven, Leuven, Belgium; k Department of Respiratory Medicine and Infectious Diseases, Hannover Medical School, Frankfurt, Germany; l Biomedical Research in End-Stage and Obstructive Lung Disease Hannover, German Center for Lung Research, Hannover, Germany; m European Reference Network on Rare and Complex Respiratory Diseases, Frankfurt, Germany; n Department of Pathophysiology and Transplantation, Università degli Studi di Milano, Italy; o Respiratory Unit and Cystic Fibrosis Center, Fondazione IRCCS Cà Granda Ospedale Maggiore Policlinico Milano, Italy; p Department of Biomedical Sciences, Humanitas University, Pieve Emanuele, Milan, Italy; q Respiratory Unit, IRCCS Humanitas Research Hospital, Rozzano, Milan, Italy

**Keywords:** antiglycopeptidolipid core IgA test, bronchiectasis, nontuberculous mycobacteria, serodiagnosis

## Abstract

**Background:**

The serum antiglycopeptidolipid core IgA antibody test has been proposed as a diagnostic tool for *Mycobacterium avium* complex pulmonary diseases. Cross-reactivity with other nontuberculous mycobacteria (NTM), including *Mycobacterium abscessus*, indicates that it may have a role as a broader screening test for nontuberculous mycobacterial pulmonary disease (NTM-PD). NTM-PD is believed to be underdiagnosed in patients with bronchiectasis.

**Research Question:**

Can the serum antiglycopeptidolipid core IgA antibody test be used to screen for NTM-PD in bronchiectasis?

**Study Design and Methods:**

Patients from the prospective European Bronchiectasis Registry (European Multicentre Bronchiectasis Audit and Research Collaboration-Bronchiectasis Research Involving Databases, Genomics and Endotyping; ClinicalTrails.gov Identifier: NCT03791086) were enrolled. Patients from the United Kingdom, Italy, Spain, Belgium, The Netherlands, and Germany were included. A control cohort of patients without any underlying lung disease also was recruited. The levels of serum IgA antibodies against the glycopeptidolipid core were measured using an enzyme immunoassay kit, and receiver operating characteristics curve analysis was conducted to evaluate the accuracy of the antibody level in screening for NTM-PD.

**Results:**

Two hundred eighty-two patients were enrolled (151 female patients [53.6%]; median age, 68 years). Median antiglycopeptidolipid core IgA antibody levels were 0.2 U/mL (interquartile range [IQR], 0.1-0.3 U/mL) in patients without NTM isolation and NTM-PD (n = 238), 0.3 U/mL (IQR, 0.2-0.4 U/mL) in patients with NTM isolation that was incompatible with the diagnosis of NTM-PD (n = 18), and 1.5 U/mL (IQR, 0.4-6.2 U/mL) in patients with NTM-PD (n = 26; *P* = .0001). Antibody levels showed excellent accuracy in identifying patients with NTM-PD (area under the receiver operating characteristic curve, 0.886; 95% CI, 0.800-0.973) in the bronchiectasis cohort and also showed excellent discrimination of patients with NTM-PD from those with NTM isolation who did not meet the diagnostic criteria for NTM-PD (0.816; 95% CI, 0.687-0.945).

**Interpretation:**

The antiglycopeptidolipid core IgA antibody demonstrated excellent efficacy in screening for NTM-PD in a large cohort of patients with bronchiectasis.

**Clinical Trial Registry:**

ClinicalTrials.gov; No.: NCT03791086; URL: www.clinicaltrials.gov

**Take-Home Points**

**Study Question:** Can the serum antiglycopeptidolipid core IgA antibody test be used to screen for nontuberculous mycobacterial pulmonary disease (NTM-PD) in bronchiectasis?

**Results:** We found that antibody levels showed excellent accuracy in identifying patients with NTM-PD in a large cohort with bronchiectasis and showed excellent discrimination between patients with NTM-PD and those with nontuberculous mycobacteria isolation who did not meet the diagnostic criteria for NTM-PD.

**Interpretation:** The antiglycopeptidolipid core IgA antibody demonstrated excellent efficacy in screening for NTM-PD in a large cohort with bronchiectasis.

The relationship between nontuberculous mycobacterial pulmonary disease (NTM-PD) and non-cystic fibrosis bronchiectasis (hereafter referred to as bronchiectasis) is well known.^1^, ^2^, ^3^ NTM-PD was determined to be the cause of bronchiectasis in 1%, 4%, and 9% of patients from European, Korean, and Australian registries, respectively^4^, ^5^, ^6^, ^7^; furthermore, approximately 13% of patients with bronchiectasis received a diagnosis of NTM-PD in a southern European bronchiectasis registry.^8^ In addition to the high prevalence of NTM-PD in bronchiectasis, the fact that NTM-PD is one of the treatable causes of bronchiectasis has led international guidelines to recommend mycobacterial sputum cultures for the initial evaluation and follow-up tests of patients with bronchiectasis.^9^^,^^10^ However, diagnostic challenges exist in some patients with bronchiectasis who have difficulty expectorating sputum and the low sensitivity and time-consuming nature of mycobacterial culture analysis.^11^ Moreover, international guidelines recommend that symptoms and radiologic features must be present to diagnose NTM-PD,^12^ but these may overlap with clinical and radiologic features of bronchiectasis without NTM-PD. Therefore, NTM-PD is believed to be underdiagnosed in patients with bronchiectasis.

To address these diagnostic challenges, an enzyme immunoassay kit that detects the IgA antibody reacting to the glycopeptidolipid core antigen of *Mycobacterium avium* complex (MAC) was developed^13^^,^^14^ and has been approved as a diagnostic tool for MAC pulmonary disease (MAC-PD) in Japan.^15^ The assay has demonstrated usefulness for diagnosing MAC-PD with a reported sensitivity of 54% to 92% and a specificity of 72% to 99% in prior studies.^14^^,^^16^, ^17^, ^18^, ^19^, ^20^ Moreover, as the assay also showed cross-reactivity with other nontuberculous mycobacteria (NTM) species, including *Mycobacterium abscessus*, which also possess glycopeptidolipid,^21^^,^^22^ and may play a role as a broader screening test for NTM-PD in patients with bronchiectasis. However, no studies have investigated its diagnostic role in a large cohort of patients with bronchiectasis. This study aimed to evaluate the clinical efficacy of the antiglycopeptidolipid core IgA antibody test for screening NTM-PD in a large bronchiectasis cohort and to assess whether the test can discriminate patients with NTM-PD from those with NTM isolation who do not meet the diagnostic criteria for NTM-PD.

## Study Design and Methods

### Study Cohorts

This study enrolled patients with bronchiectasis from the European Multicentre Bronchiectasis Audit and Research Collaboration (EMBARC)-Bronchiectasis Research Involving Databases, Genomics and Endotyping (BRIDGE) study, a prospective observational cohort study (ClinicalTrials.gov Identifier: NCT03791086). Patients with stable bronchiectasis were enrolled from 7 European centers: Fondazione IRCCS Cà Granda Ospedale Maggiore Policlinico University Hospital (Milan, Italy), Hospital Clinic of Barcelona (Barcelona, Spain), University Hospitals Leuven (Leuven, Belgium), Royal Brompton Hospital (London, United Kingdom), Amsterdam University Medical Centre (Amsterdam, The Netherlands), Hannover Medical Centre (Hannover, Germany), and Ninewells Hospital (Dundee, Scotland). All participants had a diagnosis of bronchiectasis confirmed by chest CT imaging in addition to the clinical syndrome of bronchiectasis. Patients with cystic fibrosis were excluded.

Our study was designed to enroll an enriched cohort of patients with bronchiectasis that included the subset of patients with concomitant NTM-PD, considering the low prevalence of NTM-PD in European bronchiectasis cohorts.^6^ First, from the EMBARC-BRIDGE cohort, we selected a subset of patients with bronchiectasis with a diagnosis of MAC-PD or who showed MAC isolated from sputum samples, who served as a positive control group. Second, we included patients with bronchiectasis, regardless of NTM status, with serum samples sufficient for conducting the antiglycopeptidolipid core IgA antibody analysis from the EMBARC-BRIDGE study. These patients were enrolled consecutively, using the following criterion for selection: NTM culture results were available in the case report form for the year of sampling to confirm NTM status. Therefore, we did not include patients who had not had sputum or bronchial washing or lavage samples sent for mycobacterial culture to determine NTM status. Third, control participants without underlying lung diseases also were enrolled in the PREDICT-COVID-19 cohort.^23^

This study was approved by the ethical committee in the host country (United Kingdom) and by institutional review boards or ethics committees in all countries and regions where the study was conducted. More detailed information on the cohorts is provided in our previous studies.^23^, ^24^, ^25^, ^26^, ^27^

### Definitions

NTM-PD was defined using the composite criteria of respiratory symptoms, radiographic findings, and compatible microbiologic test results, according to the joint American Thoracic Society and Infectious Diseases Society of America guidelines.^28^ Compatible microbiologic test results included: (1) the same NTM species isolated from ≥ 2 sputum cultures, (2) NTM species isolated from ≥ 1 bronchial washing or lavage, or (3) biopsy sample showing mycobacterial histopathologic features and positive culture results for NTM (or ≥ 1 sputum or bronchial wash that showed positive culture results for NTM).^3^ NTM isolation was defined as NTM grown on acid-fast bacilli culture; however, the patient did not satisfy the diagnostic criteria for NTM-PD (ie, NTM was isolated from only 1 sputum culture, with other samples showing negative results). Past NTM infection was defined as bronchiectasis in patients who had completed antibiotic treatment for NTM-PD and showed negative results on serial mycobacterial sputum cultures.

### Enzyme Immunoassay

Serum samples were collected in serum separator tubes, spun, and frozen at –80 °C at each study site and shipped to the Ninewells Hospital, University of Dundee, Dundee, Scotland. Titer of antiglycopeptidolipid core IgA antibodies using Capilla MAC Ab ELISA (TANUS Laboratories, Inc.) were measured according to the manufacturer’s instructions.^13^^,^^29^

### Statistical Analysis

Data were reported as median (interquartile range [IQR]) for continuous variables and frequencies (percentages) for categorical variables. The antiglycopeptidolipid core IgA antibody assay titers were compared using the Kruskal-Wallis test with post hoc paired comparisons using Bonferroni multiple-test correction between groups. The discriminative power of the assay was assessed by performing receiver operating characteristic curve analysis. We estimated the sensitivity, specificity, positive likelihood ratio, and negative likelihood ratio for a preset manufacturer’s cut off point (≥ 0.7 U/mL) and an optimal cut off point suggested by the present study. The optimal cut off point was determined by the Youden index (a cut off point showing the highest value calculated as: [sensitivity + specificity] – 1).^30^ All statistical analyses were performed using Stata version 16 software (StataCorp LP), and graphs were compiled using GraphPad Prism version 10.1.2 software (GraphPad Software).

## Results

### Study Population and Characteristics

**Figure 1:**
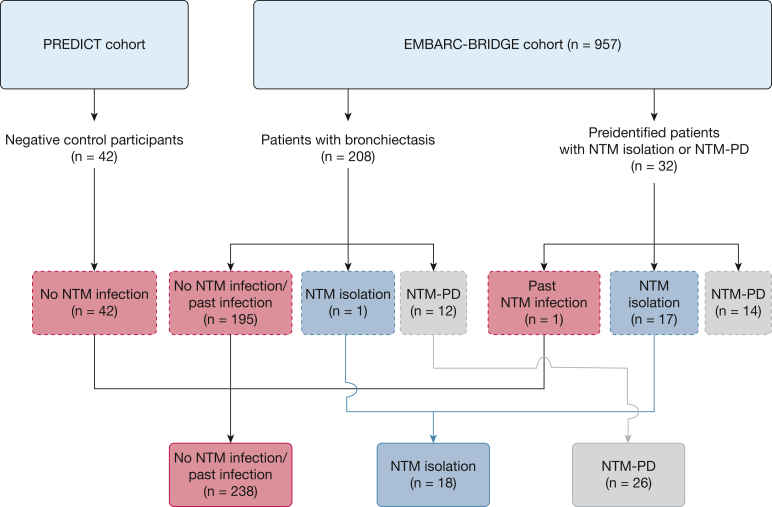
Flow diagram showing study population. BRIDGE = Bronchiectasis Research Involving Databases, Genomics, and Endotyping; EMBARC = European Multicentre Bronchiectasis Audit and Research Collaboration; NTM = nontuberculous mycobacteria; NTM-PD = nontuberculous mycobacterial pulmonary disease.

Among 957 patients with bronchiectasis enrolled in the EMBARC-BRIDGE cohort through March 2023, we initially included 32 preidentified patients with NTM isolation (see Methods) and consecutively included serum samples from a further 208 patients with bronchiectasis; among the total of 240 patients with bronchiectasis, 196 patients with bronchiectasis (81.7%) did not have NTM infection, including 3 patients with past infection, 18 patients (7.5%) had NTM isolation (all species were MAC), and 26 patients (10.8%) had NTM-PD (all MAC except 3 with *M abscessus* infection). Forty-two control participants, included as negative control participants in this study, did not have bronchiectasis, NTM isolation, or NTM-PD (Fig 1). All participants with NTM-PD demonstrated bronchiectasis on CT imaging without cavitary disease.

The median age of the patients with bronchiectasis (n = 240) was 69 years (IQR, 59-76 years), and 126 patients (52.5%) were female. Regarding comorbidities, 82 patients (34.2%), 50 patients (20.8%), and 16 patients (6.7%) had asthma, COPD, and diabetes mellitus, respectively (Table 1). The control participants were well matched for age and sex: the median age of the negative control participants (n = 42) was 68 years (IQR, 53-76 years) and 25 participants (59.5%) were female.

**Table 1:** Clinical Characteristics of Patients With Bronchiectasis

Variable	Bronchiectasis Cohort (n = 240)^a^
Age, y	69 (59-76)
Female sex	126 (52.5)
BMI, kg/m^2^	25.7 (21.7-29.4)
NTM status	
No NTM infection^b^	196 (81.7)
NTM isolation	18 (7.5)
NTM pulmonary disease	26 (10.8)
NTM species	
*Mycobacterium avium* complex	41
*Mycobacterium abscessus*	3
Comorbidities	
Asthma	82 (34.2)
COPD	50 (20.8)
Diabetes mellitus	16 (6.7)
Smoking history	
Never	140 (58.3)
Former	94 (39.2)
Current	6 (2.5)
Pulmonary function	
FEV_1_, L	1.85 (1.44-2.59)
FEV_1_, % predicted	80.0 (59.8-98.7)
FVC	3.00 (2.37-3.71)
FVC, % predicted	99.1 (81.6-113.5)
Exacerbations in the previous year	0 (0-1)
Bronchiectasis severity index	6 (4-9)

Data are presented as No., No. (%), or median (interquartile range). NTM = nontuberculous mycobacteria; NTM-PD = nontuberculous mycobacteria pulmonary disease.

a An enriched cohort of patients with bronchiectasis including the subset of patients with concomitant NTM-PD, considering a low prevalence of NTM-PD in European Bronchiectasis Cohorts.

b Three patients had past nontuberculous mycobacterial infection.

### Level of Antibody to Glycopeptidolipid Core

**Figure 2:**
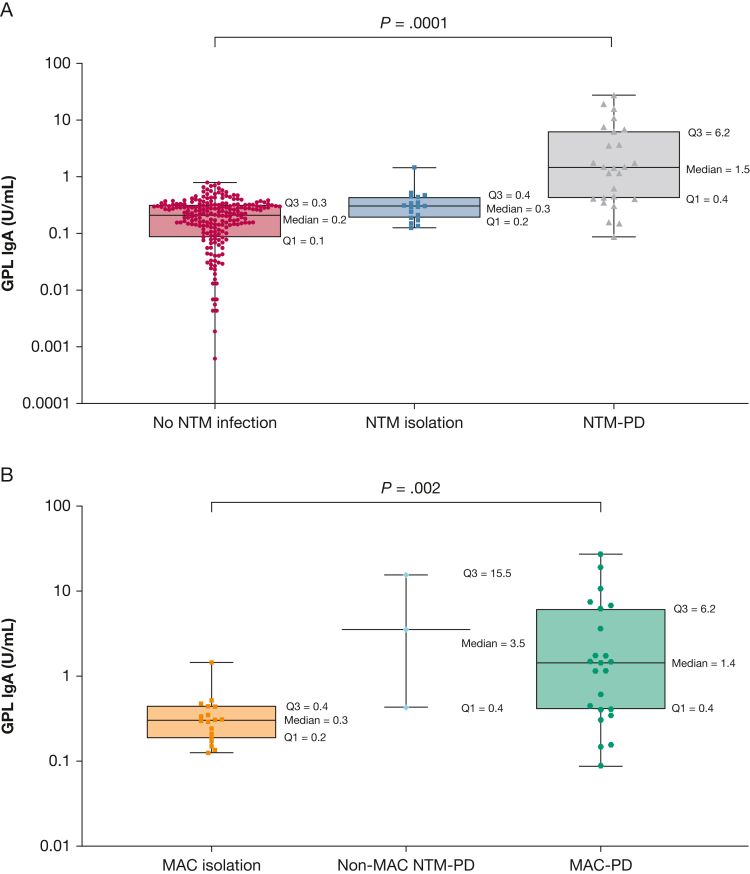
A, B, Box-and-whisker plots showing serum antiglycopeptidolipid core IgA antibody comparison among no NTM infection, NTM isolation (incompatible with the diagnosis of NTM-PD) and NTM-PD in all study participants (N = 282) (A) and MAC isolation (incompatible with the diagnosis of NTM-PD), non-MAC NTM-PD, and MAC-PD in the subset of participants with NTM isolation or NTM-PD (n = 44) (B). The whiskers are drawn from the box down to the minimum value and up to the maximum. The Kruskal-Wallis test was used to compare the titers of each study group. GPL = glycopeptidolipid; MAC = *Mycobacterium avium* complex; MAC-PD = *Mycobacterium avium* complex pulmonary disease; NTM = nontuberculous mycobacteria; NTM-PD = nontuberculous mycobacterial pulmonary disease; Q1 = first quartile; Q3 = third quartile.

In an analysis of all study participants (N = 282), antiglycopeptidolipid core IgA antibody levels were significantly higher in patients with NTM-PD (median, 1.5 U/mL; IQR, 0.4-6.2 U/mL) than in those with NTM isolation (median, 0.3 U/mL; IQR, 0.2-0.4 U/mL; *P* = .013 with Bonferroni correction) and significantly higher than in those without NTM isolation and NTM-PD (median, 0.2 U/mL; IQR, 0.1-0.3 U/mL; *P* < .0001 with Bonferroni correction; *P* = .0001 comparing the 3 groups) (Fig 2). Among participants without NTM isolation and NTM-PD (n = 238), no significant difference was found in antiglycopeptidolipid core IgA antibody level between negative control participants and patients with bronchiectasis (*P* = .83).

Furthermore, when analyzing only 44 patients with NTM isolation or NTM-PD, both patients with MAC-PD (n = 23; median, 1.4 U/mL; IQR, 0.4-6.2 U/mL; *P* = .0003 with Bonferroni correction) and those with non-MAC NTM-PD (n = 3; median, 3.5 U/mL; IQR, 0.4-15.5 U/mL; *P* = .008 with Bonferroni correction) showed significantly higher antiglycopeptidolipid core IgA antibody levels than in those with MAC isolation (n = 18; median, 0.3 U/mL; IQR, 0.2-0.4 U/mL; *P* = .002 comparing the 3 groups) (Fig 2).

### Discriminative Performance and Cut Off Points

**Figure 3:**
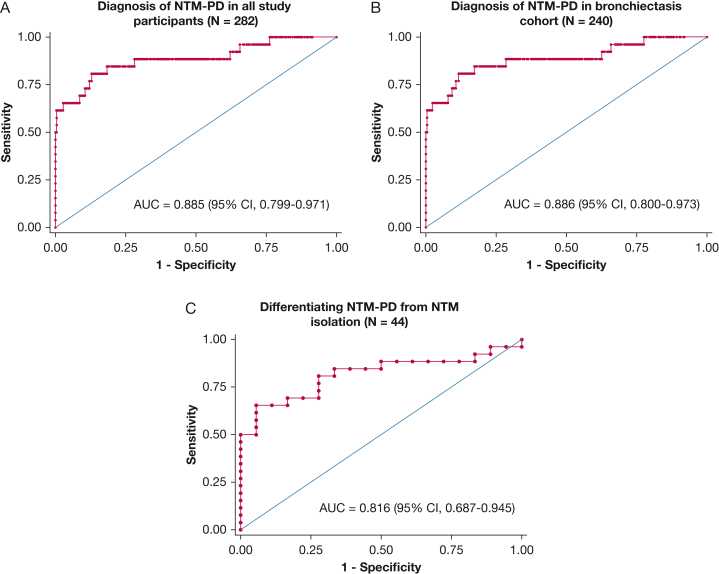
A-C, Graphs showing receiver operating characteristic curves of serum antiglycopeptidolipid core IgA antibody for diagnosing NTM-PD in all study participants (N = 282) (A), diagnosing NTM-PD in bronchiectasis cohort (n = 240) (B), and differentiating NTM-PD from NTM-isolation (n = 44) (C). AUC = area under the receiver operating characteristic curve; NTM = nontuberculous mycobacteria; NTM-PD = nontuberculous mycobacterial pulmonary disease.

In receiver operating characteristic curve analyses, the antiglycopeptidolipid core IgA antibody level demonstrated excellent performance in diagnosing NTM-PD in all study participants (n = 282; area under the curve, 0.885; 95% CI, 0.799-0.971), diagnosing NTM-PD in the bronchiectasis cohort (n = 240; area under the receiver operating characteristic curve, 0.886; 95% CI, 0.800-0.973), and in differentiating NTM-PD from NTM isolation (n = 44; area under the receiver operating characteristic curve, 0.816; 95% CI, 0.687-0.945) (Fig 3).

When identifying NTM-PD in the bronchiectasis cohort (n = 240), antiglycopeptidolipid core IgA antibody level showed 61.5% sensitivity, 98.6% specificity, and 43.9 positive likelihood ratio at the manufacturer’s cut off (0.7 U/mL) and 80.8% sensitivity, 88.3% specificity, and 6.9 positive likelihood ratio at an optimal cut off suggested in this study (0.4 U/mL). Additionally, when discriminating NTM-PD from NTM isolation (n = 44), the antiglycopeptidolipid core IgA antibody level showed 65.4% sensitivity, 94.4% specificity, and 11.8 positive likelihood ratio at the manufacturer’s cut off (0.7 U/mL), which also was the optimal cut off suggested in this study (Table 2).

**Table 2:** Discriminative Performance of Serum Antiglycopeptidolipid Core IgA Antibody at Manufacturer’s Cut Off and Optimal Cut Off Suggested in This Study

Variable	Cut Off	Glycopeptidolipid IgA, U/mL	Sensitivity, %	Specificity, %	PPV, %	NPV, %	PLR	NLR
NTM-PD screening in bronchiectasis cohort (n = 240)	Manufacturer’s cut off	≥ 0.7	61.5	98.6	84.2	95.5	43.9	0.4
	Optimal cut off^a^	≥ 0.4	80.8	88.3	45.5	97.4	6.9	0.2
NTM-PD differentiation from NTM isolation (n = 44)	Manufacturer’s cut off	≥ 0.7	65.4	94.4	94.4	65.4	11.8	0.4
	Optimal cut off^a^	≥ 0.7	65.4	94.4	94.4	65.4	11.8	0.4

NLR = negative likelihood ratio; NPV = negative predictive value; NTM = nontuberculous mycobacteria; NTM-PD = nontuberculous mycobacterial pulmonary disease; PLR = positive likelihood ratio; PPV = positive predictive value.

a Determined by Youden’s index (sensitivity + specificity – 1).

## Discussion

The serum antiglycopeptidolipid core IgA antibody test showed excellent accuracy in identifying patients with NTM-PD in a large cohort with bronchiectasis and also showed excellent discrimination between patients with NTM-PD and those with NTM isolation who did not meet the joint American Thoracic Society and Infectious Diseases Society of America diagnostic criteria for NTM-PD. When identifying NTM-PD in the cohort with bronchiectasis, the optimal cut off suggested in this study increased the sensitivity of the assay (sensitivity, 81%; specificity, 88%) compared with the predefined cut off (sensitivity, 62%; specificity, 99%). However, when discriminating NTM-PD from NTM isolation, the optimal cut off suggested in this study was identical to the predefined cut off value.

Notably, to our knowledge, this is the first study to investigate the serum antiglycopeptidolipid core IgA antibody test and demonstrate its efficacy in a large cohort with bronchiectasis, although previous studies included a relatively small number of patients with bronchiectasis as a control group.^15^^,^^20^^,^^29^ In addition to identifying NTM-PD in patients with newly diagnosed bronchiectasis, the assay could be useful during the follow-up of patients with bronchiectasis. Considering its high specificity (99% at ≥ 0.7 U/mL of cut off), the assay may serve successfully as a rule-in diagnostic test to identify patients with bronchiectasis with NTM-PD who may require a more intensified diagnostic workup for NTM-PD. In people with bronchiectasis, differentiating the symptoms and radiologic features between NTM-PD and baseline bronchiectasis would be very difficult because considerable overlap exists between the two conditions in terms of both clinical and radiologic features. Using high serum antiglycopeptidolipid core IgA antibody titers may strengthen the decision to diagnose NTM-PD and start antimycobacterial treatment when necessary. Importantly, our results are in line with previous validation studies using this assay that found it to have high specificity, but relatively low sensitivity.^15^^,^^31^^,^^32^ This means that when the results are positive, it makes NTM-PD highly likely, but that negative results do not exclude the presence of NTM-PD definitively. Thus, like any diagnostic assay, it is likely to be useful only when combined with other clinical or diagnostic methods to exclude a diagnosis of NTM-PD.

Interestingly, the IgA assay also was useful in identifying patients with *M abscessus* in this study. Although the assay was developed for diagnosing MAC-PD, it also shows cross-reactivity with many other NTM species that possess glycopeptidolipid on their cell walls, including *M abscessus*, *Mycobacterium fortuitium*, *Mycobacterium chelonae*, and *Mycobacterium simiae*.^22^^,^^33^ Cross-reactivity is a pitfall of the assay when diagnosing MAC-PD. However, it may be beneficial if the assay is used as a broader screening test for NTM-PD during the initial investigation and follow-up of patients with bronchiectasis. Further studies are needed to determine how this test can be incorporated into the clinical care of patients with bronchiectasis to screen and diagnose NTM-PD.

Another notable finding of this study was that the assay showed excellent specificity of 99%, but relatively low sensitivity of 62% for identifying NTM-PD in bronchiectasis cohort at the manufacturer’s cut off (0.7 U/mL), in contrast to previous studies from Korea (sensitivity, 85%; specificity, 100%) and Japan (sensitivity, 84%; specificity, 100%).^21^^,^^29^ The different sensitivities may be the result of geographic and ethnic differences or different characteristics of the cohorts, because 2 previous studies conducted in the United States also showed lower sensitivity (48% and 52%); the sensitivity was enhanced by setting lower cut off points,^15^^,^^31^ which was similar to our findings (sensitivity of 81% and specificity of 88% at a cut off of 0.4 U/mL). Furthermore, factors associated with lower glycopeptidolipid antibody levels include immunocompromised diseases such as malignancy and macrolide monotherapy,^32^^,^^34^ which warrants the caution of potentially higher false-negative rates and the necessity of selecting different optimal cut off points in these populations.

To the best of our knowledge, this study is the first to investigate the role of serum antiglycopeptidolipid core IgA antibodies in screening for NTM-PD in a large cohort with bronchiectasis. However, this study has some limitations. First, we used an enrichment design in which patients with known NTM isolation were selected because NTM is relatively uncommon in European patients with bronchiectasis. Although this enhances the power to evaluate the test using a moderate sample size, the pickup rate and cost-effectiveness of a screening strategy using this test in an unselected fashion has not been established. Second, because the glycopeptidolipid is not located on the surface of the *Mycobacterium kansasii* cell wall,^22^ our results should be applied cautiously in countries where the prevalence of *M kansasii* is high. Third, despite the efficacy of identifying NTM-PD regardless of the NTM species in this study, only 3 patients with non-MAC NTM-PD were included. Fourth, this study was conducted in 6 European countries with mostly White populations. Therefore, future studies should be conducted in other geographical regions with different NTM prevalence and ethnic groups to validate our findings. Finally, this study lacked data from longitudinally exploring change in IgA antibody levels over time among those undergoing watchful waiting or treatment.

## Interpretation

The serum antiglycopeptidolipid core IgA antibody test showed excellent accuracy in identifying patients with NTM-PD in a large cohort with bronchiectasis and also showed excellent discrimination between patients with NTM-PD and those with NTM isolation who did not meet the diagnostic criteria for NTM-PD.

## Funding/Support

This research was funded by the European Respiratory Society through the EMBARC3 consortium. EMBARC3 is supported by project partners Armata, AstraZeneca, Boehringer Ingelheim, Chiesi, CSL Behring, Glaxosmithkline, Grifols, Insmed, Janssen, Lifearc, and Zambon. J. D. C. is supported by the Asthma and Lung UK Chair of Respiratory Research.

## Financial/Nonfinancial Disclosures

The authors have reported to *CHEST* the following: H. C. reports grant from the Basic Science Research Program of the Korean Ministry of Education (grant no. 2021R1I1A3052416); and lecture fees from Boryung Pharmaceutical Co. and Kolon Pharma and Abbott. M. Shteinberg reports consulting fees from GSK, Boehringer Ingelheim, Kamada, Medison, and Zambon; payment or honoraria for lectures, presentations, speakers bureaus, manuscript writing, or educational events from Insmed, Boehringer Ingelheim, GSK, AstraZeneca, Teva, Novartis, Kamada, and Sanofi; support for attending meetings, travel, or both from Novartis, Actelion, Boehringer Ingelheim, GSK, and Rafa; participation on a data safety monitoring board or advisory board for Bonus Therapeutics, Israel; leadership or fiduciary role in other board, society, committee, or advocacy group, paid or unpaid for EMBARC Management, Israel Pulmonology Society Board, Israel Society for TB and Mycobacterial Diseases; receipt of equipment, materials, drugs, medical writing, gifts, or other services from Trudell Medical Int.; and other financial or nonfinancial interests including associate editor, *American Journal of Respiratory and Critical Care Medicine*. E. P. reports grants or contracts from any entity from Grifols; consulting fees from Insmed, Bayer, Chiesi, and Zambon; payment or honoraria for lectures, presentations, speakers bureaus, manuscript writing, or educational events from Bayer, Chiesi, Grifols, GlaxoSmithKline, Insmed, Menarini, and Zambon; and support for attending meetings, travel, or both from Insmed, Pfizer, and Moderna. P. C. G. reports payment or honoraria for lectures, presentations, speakers bureaus, manuscript writing, or educational events from Insmed, GSK, and Chiesi; support for attending meetings, travel, or both from Chiesi; and participation in a data safety monitoring board or advisory board for Boehringer, GSK, and Pfizer. F. B. reports grants or contracts from any entity from AstraZeneca, Chiesi, and Insmed; consulting fees from Menarini; and payment or honoraria for lectures, presentations, speakers bureaus, manuscript writing, or educational events from AstraZeneca, Chiesi, GSK, Guidotti, Grifols, Insmed, Menarini, Novartis, OM Pharma, Pfizer, Sanofi, Viatris, Vertex, and Zambon. A. S. reports consulting fees from Spirovant and Translate Bio; payment or honoraria for lectures, presentations, speakers bureaus, manuscript writing, or educational events from Translate Bio, Ethris, and Insmed; leadership or fiduciary role in other board, society, committee, or advocacy group, paid or unpaid for European Respiratory Society Clinical Research Collaborations (EMBARC, Better Experimental Approaches to Treat Primary Ciliary Dyskinesia [BEAT-PCD], AntiMicrobial Resistance [AMR]). S. A. reports grants or contracts from any entity from Insmed Incorporated, Chiesi, Fisher and Paykel, and GSK; royalties or licences from McGraw Hill; consulting fees from Insmed Incorporated, Insmed Italy, Insmed Ireland Ltd, Zambon Spa, AstraZeneca UK Ltd., AstraZeneca Pharmaceutical LP, CSL Behring GmbH, Grifols, Fondazione Internazionale Menarini, Moderna, Chiesi, MCD Italis SrL, Brahms, Physioassist SAS, and GlaxoSmithKline Spa; payment or honoraria for lectures, presentations, speakers bureaus, manuscript writing, or educational events from GlaxoSmithKline Spa, Thermofisher Scientific, Insmed Italy, Insmed Ireland, Zambon, and Fondazione Internazionale Menarini; participation on a data safety monitoring board or advisory board from Insmed Incorporated, Insmed Italy, AstraZeneca UK Ltd., and MSD Italia Srl. C. S. H. reports payment or honoraria for lectures, presentations, speakers bureaus, manuscript writing, or educational events from 30 Technology, CSL Behring, Chisi, Insmed, Janssen, LifeArc, Meiji, Mylan, Pneumagen, Shionogi, Vertex, and Zambon. F. C. R. reports grants or contracts from any entity from German Center for Lung Research (DZL), German Center for Infection Research (DZIF), IMI (EU / EFPIA), and iABC Consortium (incl. Alaxia, Basilea, Novartis, and Polyphor), Mukoviszidose Institute, Novartis, Insmed Germany, Grifols, Bayer, and InfectoPharm; consulting fees from Parion, Grifols, Zambon, Insmed, and Helmholtz-Zentrum für Infektionsforschung; payment or honoraria for lectures, presentations, speakers bureaus, manuscript writing, or educational events from I!DE Werbeagentur GmbH, Interkongress GmbH, AstraZeneca, Insmed, Grifols, and Universitätsklinikum Frankfurt am Main; payment for expert testimony from Social Court Cologne; support for attending meetings, travel, or both from German Kartagener Syndrome and Primary Ciliary Dyskinesia Patient Advocacy Group Mukoviszidose e.V.; participation on a data safety monitoring board or advisory board for Insmed, Grifols and Shionogi; leadership or fiduciary role in other board, society, committee, or advocacy group, paid or unpaid, as coordinator of the European Reference Network Respiratory Diseases (ERN-LUNG) Bronchiectasis Core Network, chair of the German Bronchiectasis Registry Prospective German Non-cystic fibrosis Bronchiectasis Patient Registry (PROGNOSIS), member of the SteerCo of the European Bronchiectasis Registry EMBARC, member of the SteerCo of the European Nontuberculous Mycobacterial Pulmonary Disease Registry EMBARC-NTM, cospeaker for the Medical Advisory Board of the German Kartagener Syndrome and PCD Patient Advocacy Group, speaker of the Respiratory Infections and TB Group of the German Respiratory Society, speaker of the Cystic Fibrosis Group of German Respiratory Society (DGP), primary investigator of the German Center for Lung Research, member of the Protocol Review Committee of the Clinical Trial Network for Primary Ciliary Dyskinesia (PCD-CTN), and member of Physician Association of the German Cystic Fibrosis Patient Advocacy Group; and other financial or nonfinancial interests with AstraZeneca, Boehringer Ingelheim, Celtaxsys, Corbus, Insmed, Novartis, Parion, University of Dundee, Vertex, and Zambon. M. R. L. reports consulting fees from Armata, 30T, AstraZeneca, Parion, Insmed, Chiesi, Zambon, Electromed, Recode, AN2, and Boehringer Ingelheim; payment or honoraria for lectures, presentations, speakers bureaus, manuscript writing, or educational events from Insmed; leadership or fiduciary role in other board, society, committee or advocacy group, paid or unpaid, as ERS Infection Group Chair. N. L. reports leadership or fiduciary role in other board, society, committee or advocacy group, paid or unpaid, as a founder and chair of the Belgian NTM Clinical Advisory Board and a chair of Non-tuberculous Mycobacteria Network European Trials group (NTM-NET). J. D. C. reports grants or contracts from any entity from AstraZeneca, Boehringer Ingelheim, Genentech, Gilead Sciences, GlaxoSmithKline, Grifols, Insmed, LifeArc, and Novartis; and consulting fees from AstraZeneca, Chiesi, GlaxoSmithKline, Insmed, Grifols, Novartis, Boehringer Ingelheim, Pfizer, Janssen, Antabio, and Zambon. None declared (C. H., Z. E., M. Shuttleworth, T. W., M. B. L.).
